# Quantification and Analysis of Lung Involvement by Artificial Intelligence in Patients with Progressive Pulmonary Fibrosis Treated with Nintedanib

**DOI:** 10.3390/medicina61091646

**Published:** 2025-09-11

**Authors:** Caterina Battaglia, Corrado Pelaia, Chiara Lupia, Alessia Mondelli, Francesco Turco, Paolo Zaffino, Carlo Cosentino, Francesco Manti, Giuliana Conti, Nicola Montenegro, Antonio Maiorano, Girolamo Pelaia, Pasquale Romeo, Domenico Laganà

**Affiliations:** 1 Department of Experimental and Clinical Medicine, University Magna Graecia, 88100 Catanzaro, Italy; caterina.battaglia@unicz.it (C.B.); mondelli.alessia@gmail.com (A.M.); francesco.turco002@studenti.unicz.it (F.T.); p.zaffino@unicz.it (P.Z.); carlo.cosentino@unicz.it (C.C.); manti.fra@gmail.com (F.M.); giuliana.conti92@gmail.com (G.C.); domenico.lagana@unicz.it (D.L.); 2 Department of Medical and Surgical Science, University Magna Graecia, 88100 Catanzaro, Italy; 3 Department of Health Sciences, University Magna Graecia, 88100 Catanzaro, Italy; chiaralupia1996@gmail.com (C.L.); n.montenegro@hotmail.it (N.M.); antoniomaiorano95@gmail.com (A.M.); pelaia@unicz.it (G.P.); pasqualeromeo63@libero.it (P.R.)

**Keywords:** artificial intelligence, lung analysis, segmentation, progressive pulmonary fibrosis, nintedanib

## Abstract

*Background and Objectives:* Progressive pulmonary fibrosis (PPF) presents significant clinical challenges due to irreversible lung damage and declining respiratory function. Nintedanib has demonstrated antifibrotic effects, yet there is a lack of sensitive tools to assess treatment efficacy quantitatively. This study evaluated the potential of artificial intelligence (AI)-powered quantitative computed tomography (QCT) to monitor lung changes and predict treatment outcomes in patients with PPF undergoing nintedanib therapy. *Materials and Methods:* This retrospective study analysed 37 patients diagnosed with PPF who were treated with nintedanib for one year. AI-powered QCT was performed using the 3D Slicer software version 5.2.2, which quantified lung infiltration, collapse, and vessel volumes. These data were then correlated with pulmonary function tests. Receiver operating characteristic (ROC) analysis was used to assess baseline AI-powered QCT predictors for progression. *Results:* AI-powered QCT demonstrated a significant reduction in post-treatment right lung infiltration (5.56 ± 3.08 cm^3^ to 4.88 ± 2.77 cm^3^, *p* = 0.041), whereas total lung infiltration decreased non-significantly. Functional parameters, including forced vital capacity (FVC) and diffusion capacity for carbon monoxide (DLCO), showed no significant changes. ROC analysis identified a baseline infiltrated lung volume greater than 21.90% as predictive of continued disease progression (AUC = 0.767; sensitivity, 91.70%; specificity, 68.00%). *Conclusions:* AI-powered QCT identified diverse parenchymal responses to nintedanib in PPF and showed preliminary prognostic value for clinical trajectory. Imaging biomarkers enhance functional measures and may reveal early treatment effects. Prospective, multicentre validation is necessary to confirm usefulness and establish actionable thresholds for clinical application.

## 1. Introduction

Progressive pulmonary fibrosis (PPF) presents a significant clinical challenge, characterised by the relentless and irreversible scarring of lung tissue, ultimately leading to a progressive decline in respiratory function [1]. Historically, fibrosing lung diseases were categorised and managed primarily based on their underlying aetiology, such as idiopathic pulmonary fibrosis (IPF), connective tissue disease-associated interstitial lung disease (CTD-ILD), or chronic hypersensitivity pneumonitis (HP). However, a critical paradigm shift has occurred in recent years, moving from this aetiology-centric view to the recognition of a common clinical and biological trajectory—the progressive fibrosing phenotype [2]. This phenotype is characterised by a self-sustaining fibrotic process that, once initiated, may continue to progress independently of the original trigger, sharing common pathobiological pathways across different underlying diagnoses [3]. This conceptual evolution is of profound clinical importance, as it provides the rationale for applying targeted antifibrotic therapies to a broader spectrum of patients exhibiting this progressive behaviour [4]. Progressive pulmonary fibrosis, as a chronic form of fibrosing interstitial pneumonia, represents the most prevalent type of ILD globally, affecting an estimated 5 million people [5]. While IPF is the archetypal and most common form, a significant proportion of patients with other non-IPF ILDs also develop this progressive phenotype. Estimates suggest that between 13% and 53% of patients with non-IPF fibrosing ILDs will experience disease progression, with some cohort studies indicating that 18% to 32% of patients meet progression criteria within several years of their initial diagnosis [6]. Data from Europe and the United States suggest a prevalence of up to 28 cases per 100,000 people. This figure is expected to rise with the improvement of diagnostics and an ageing population [7]. The demographic profile of PPF mirrors that of IPF, with a higher incidence in men than in women and an average age of onset around 70 years [8].

A confluence of clinical, physiological, and radiological factors has been identified that increases the risk of developing a progressive course. These risk factors, consistent across multiple ILD subtypes, include older age, male sex, a history of cigarette smoking, lower baseline pulmonary function, as measured by forced vital capacity (FVC) and alveolar-capillary diffusion capacity for carbon monoxide (DLCO), and, most notably, the presence of a usual interstitial pneumonia (UIP) pattern on high-resolution computed tomography (HRCT) imaging [9]. The latter is the preferred diagnostic tool, enabling clinicians to assess the distribution and extent of lung damage accurately. Key radiological markers of PPF, such as honeycombing, traction bronchiectasis, and reticulations, often display a subpleural and basal predominance. Traditionally, radiologists visually assess fibrosis on CT images and monitor its progression; however, a validated quantitative method for evaluating pulmonary fibrosis has not yet been established. Recent advances in computational methods now enable the use of quantitative CT analysis, where computed algorithms measure the extent of fibrotic lung tissue as a surrogate endpoint in research and a method for tracking treatment outcomes [10,11,12]. The prognostic potential of these new tools, including artificial intelligence (AI)-powered quantitative computed tomography (QCT) with segmentation software, lies in their ability to assess well-aerated versus pathological lung regions quantitatively. AI-driven systems automatically segment CT images using pre-defined Hounsfield Unit (HU) thresholds, which differentiate between tissue densities [13]. This automated process divides the lung volume into three equal zones and selects pixels with HU values between −200 and −1024, after which radiologists review the segmented images to ensure accuracy. Additional parameters, such as those derived from attenuation histograms, can also be calculated [14,15]. Such quantitative indexes are becoming increasingly important for evaluating the extent of ILDs and tracking disease progression under different therapeutic regimens.

The approval of the anti-fibrotic agents nintedanib and pirfenidone has marked a significant shift in PPF management [16,17]. Although neither drug halts nor reverses disease progression, they have been shown to slow it down. Nintedanib, initially developed as an anti-cancer agent, is a tyrosine kinase inhibitor that interferes with fibrosis-related processes, reducing extracellular matrix secretion and lung inflammation [18]. The present study focuses on evaluating the efficacy of nintedanib in slowing the progression of PPF, utilising quantitative CT-based scores and correlations with DLCO and FVC values. The 3D Slicer software was employed to conduct lung segmentation and image analysis on CT scans; subsequently, the functional respiratory parameters were correlated with the calculated scores. The primary aim of the study was to evaluate the effects of nintedanib on radiological scores and lung function parameters, with the goal of determining the role of baseline 3D-Slicer segmentation parameters in predicting the number of patients with PPF who no longer meet progression criteria after one year of treatment with nintedanib.

## 2. Materials and Methods

This retrospective study was conducted collaboratively by the Radiology and the Respiratory Units of “Magna Græcia” University Hospital in Catanzaro, Italy. The inclusion criteria were based on the diagnosis of PPF according to the American Thoracic Society (ATS)/European Respiratory Society (ERS)/Japanese Respiratory Society (JRS)/Latin American Thoracic Society (LATS) clinical practice guideline [19]. Diagnoses were confirmed through a multidisciplinary approach involving both radiologists and pulmonologists. Exclusion criteria include comorbid lung disease, lack of follow-up, poor-quality CT scans, and prior antifibrotic therapy. The patient assessment period spanned from 2022 to 2024. The following data were collected: age, sex, treatment initiation date, HRCT-based radiological evaluations, and pre- and post-treatment functional parameters. Patients were selected from the electronic radiology information system (RIS) and picture archiving and communication system (PACS). To facilitate the analysis, a quantitative scoring system was developed to assess the extent of pulmonary fibrosis, and these scores were correlated with pulmonary function test results using the 3D Slicer software.

### 2.1. Analysis Using 3D Slicer

**Figure 1:**
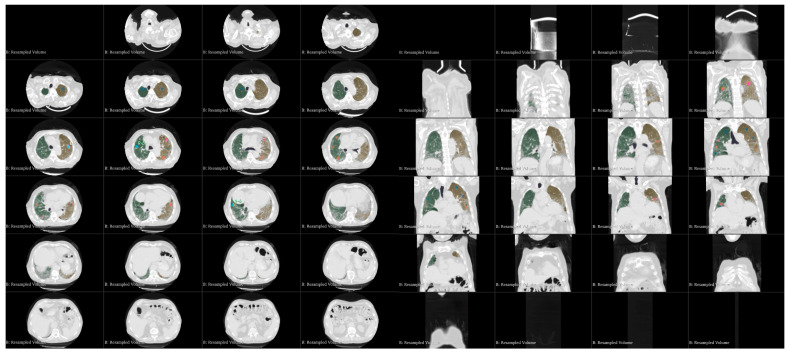
Example of a “LungCTAnalyzer extension” report, employed to quantify the volumes of infiltrated and collapsed lung tissue and intrapulmonary vessels. Segmentation of the left lung is represented in orange; segmentation of the right lung is represented in green.

**Figure 2:**
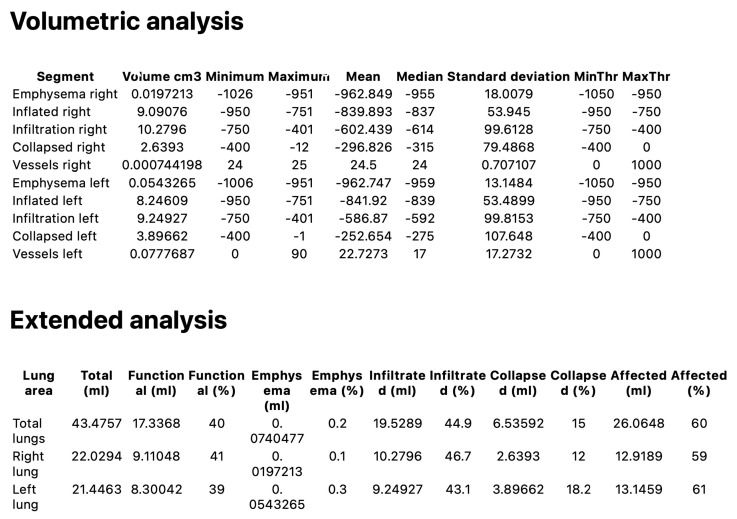
Example of a “LungCTAnalyzer results” report from an enrolled patient, including volumetric and extended analysis. Segments are created based on their Hounsfield units, using predefined threshold ranges. Functional versus affected lung volumes are shown. “Infiltration” and “Collapsed” currently include perivascular/bronchial tissues and will also never be zero.

Quantitative analysis was performed using 3D Slicer (3D Slicer, https://www.slicer.org; version 5.2.2, Boston, MA, USA), an open-source software designed for medical image processing and visualisation. Specifically, we utilised the features provided by the “Chest Imaging Platform” and “LungCTAnalyzer” extensions. DICOM (Digital Imaging and Communication in Medicine) images were acquired, and lung masks were automatically segmented using the “LungCTSegmenter” extension [20,21,22]. All automated segmentations (lung contours and class maps) underwent a two-reader quality assurance process. Two thoracic radiologists with several years of experience, blinded to clinical data and imaging time points, independently reviewed all AI-generated outputs. Minor inaccuracies—most often along the juxtacardiac left lower lobe border, around the perihilar vessels, and at pleural or basal interfaces—were corrected in 3D Slicer’s Segment Editor. Any discrepancies were resolved during a joint consensus review, and only masks finalised by consensus were exported for analysis. To characterise the dataset and cross-validate the QCT metrics, the readers also recorded a semi-quantitative visual estimate of the percentage of normal lung (per lung and whole lung). As prespecified, this visual score was used solely for descriptive quality control and was not included in hypothesis testing. The segmentation process involved manually placing three points in both axial and coronal views within the right and left lungs, as well as one point in the trachea. All lung masks were visually inspected, with minor manual adjustments made using the “Segment Editor” tools when necessary. Following this, the lung volume was segmented from the surrounding tissues and subdivided into equal sections. The “LungCTAnalyzer” extension was used to quantify the volumes of infiltrated and collapsed lung tissue, expressed both in millilitres and as percentages. In addition, intrapulmonary vessels were segmented. A demonstration of this process, referred to as an enrolled patient, is shown in Figure 1. The following HU (Hounsfield Unit) ranges were used to classify each tissue type: emphysema [−1050 to −950 HU], inflated [−950 to −750 HU], infiltration [−750 to −400 HU], and collapsed [−400 to 0 HU]. For vessels, the range was 0 to 1000 HU. After segmentation, the readers reviewed the images, and inaccurate segmentations were excluded. Subsequently, the HRCT attenuation histogram of the lungs was generated, and specific quantitative parameters were automatically calculated. QCT indices have become valuable tools for standardised analysis of ILDs. This analysis was performed for patients treated with nintedanib before and after treatment. HAA% reflects the presence of parenchymal abnormalities such as ground-glass opacity and reticulation. This was calculated as the percentage of the total lung volume with attenuation values between 250 HU and 600 HU. Volumetric analysis and extended analysis were assessed for total lungs, right lungs, and left lungs. An example of the “Lung CT Analyzer Results” report from an enrolled patient is shown in Figure 2.

### 2.2. Lung Function Tests

Spirometry and body plethysmography were performed using the MasterScreen pulmonary function testing system and a MasterScreen Body (Jaeger-Viasys; CareFusion, Höchberg, Germany), following ATS/ERS guidelines [23]. Residual volume (RV), forced vital capacity (FVC), forced expiratory volume in 1 s (FEV_1_), peak expiratory flow (PEF), and forced mid-expiratory flow between 25% and 75% of FVC (FEF_25–75_) were evaluated at baseline and after 12 months of treatment with nintedanib. Moreover, diffusion lung capacity was also assessed, as corrected single-breath DLCO values were measured.

### 2.3. Statistical Analysis

The mean ± standard deviation (SD) was used for normally distributed data, while the median value with interquartile range (IQR) was used for skewed data distributions. Based on normality, parametric or non-parametric tests were selected. Data normality was evaluated through Anderson–Darling and Kolmogorov–Smirnov tests. When appropriate, the Wilcoxon signed-rank test and the paired *t*-test were performed to compare variables. Fisher’s exact test was conducted to analyse categorical variables. The overall population was divided into two groups based on the persistence of progression criteria after one year of treatment with nintedanib, defined as two of the following three criteria occurring within the past year with no alternative explanation: (1) worsening respiratory symptoms; (2) physiological evidence of disease progression (absolute decline in FVC > 5% predicted within 1 yr of follow-up, absolute decline in DLCO > 10% predicted within 1 year of follow-up); and (3) radiological evidence of disease progression (increased extent or severity of traction bronchiectasis and bronchiolectasis, new ground-glass opacity with traction bronchiectasis, new fine reticulation, increased extent or increased coarseness of reticular abnormality, new or increased honeycombing, increased lobar volume loss). The accuracy of baseline 3D Slicer parameters as predictors of pulmonary fibrosis stabilisation during nintedanib treatment, considered as categorical variables, was evaluated by constructing a receiver operating characteristic (ROC) curve. A *p*-value of less than 0.05 (two-tailed) was deemed statistically significant. Statistical analyses and figures were generated using Prism Version 10.3.0 software (GraphPad Software Inc., San Diego, CA, USA) and Jamovi Version 2.6.26 (The Jamovi Project, Sydney, Australia).

### 2.4. Ethical Statement

This observational study adhered to the standards of Good Clinical Practice (GCP) and the principles outlined in the Declaration of Helsinki. Furthermore, informed consent was obtained from all patients. Our investigation was conducted by the guidelines provided by the local Ethical Committee of the Calabria Region (Catanzaro, Italy; document no. 118, dated 9 April 2024).

## 3. Results

Forty-five patients with PPF in treatment with nintedanib were enrolled, and thirty-seven (82.22%) completed one year of treatment. Among the 37 patients included in this study, 28 (75.68%) were male. Their mean age was 73.62 ± 7.04 years, and the mean body mass index (BMI) was 26.53 ± 3.26 kg/m^2^. Twenty-seven (72.97%) of the enrolled patients were current or previous smokers.

A significant reduction was observed in right lung infiltration, decreasing from 5.56 ± 3.08 cm^3^ to 4.88 ± 2.77 cm^3^ (*p* = 0.041). Total infiltration also reduced from 10.30 ± 5.11 mL to 9.50 ± 4.73 mL, although this was not statistically significant (*p* = 0.199). Collapsed areas showed no significant change: collapsed right remained stable (1.79 ± 1.24 cm^3^ to 1.77 ± 1.61 cm^3^, *p* = 0.919) and collapsed left slightly increased from 1.62 ± 1.50 cm^3^ to 1.77 ± 1.73 cm^3^ (*p* = 0.466). Vessel volumes did not change significantly: vessels right increased marginally from 0.28 ± 0.27 cm^3^ to 0.33 ± 0.54 cm^3^ (*p* = 0.483) and vessels left from 0.21 ± 0.27 cm^3^ to 0.36 ± 0.70 cm^3^ (*p* = 0.174). Changes in volumetric analysis parameters and extended analysis values are detailed in Table 1 and Table 2, respectively.

During the same period, RV did not show statistically significant changes, varying from 1.49 ± 0.56 L to 1.29 ± 0.63 L (*p* = 0.104). FVC and DLCO did not show significant changes, varying from 2.58 ± 1.06 L to 2.50 ± 0.82 L (*p* = 0.469) and from 3.26 ± 1.22 mmol/min/kPa to 2.76 ± 1.13 mmol/min/kPa (*p* = 0.097), respectively. Lung functional parameters before and after treatment with nintedanib are detailed in Table 3.

To identify potential predictors of radiologic response, Pearson correlation analyses were conducted between the percentage change in infiltration (Δ infiltration) and all available clinical, radiologic, and functional parameters. The most significant predictors were baseline imaging features. Strong negative correlations were found with pre-treatment total infiltration (mL) (r = −0.468, *p* = 0.004), pre-treatment right infiltration (mL) (r = −0.454, *p* = 0.005), and pre-treatment right infiltrated (%) (r = −0.444, *p* = 0.006), indicating that greater baseline infiltration was associated with an increased worsening during treatment. Additional significant correlations were observed with pre-treatment left infiltration (mL) (r = −0.422, *p* = 0.009) and pre-treatment left infiltrated (%) (r = −0.402, *p* = 0.014).

To evaluate the predictive role of spirometric parameters on radiologic response, we analysed the association between Δ infiltration (%) and a comprehensive panel of lung function metrics. Correlation analysis identified post-treatment FEF_25–75_ (%) (r = −0.469, *p* = 0.041) and pre-treatment FVC (%) (r = −0.467, *p* = 0.032) as the strongest negative correlates with Δ infiltration (%), followed by post-treatment FVC (%) (r = −0.420, *p* = 0.119) and pre-treatment DLCO (%) (r = −0.417, *p* = 0.122).

**Figure 3:**
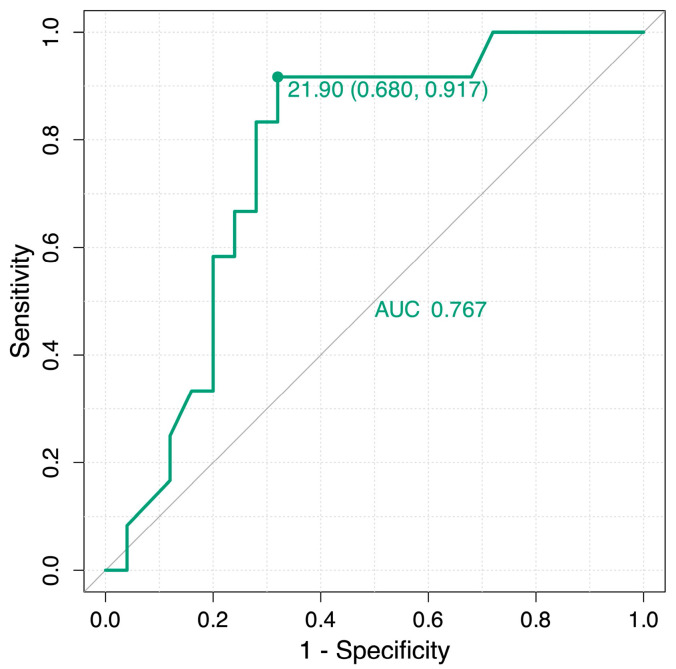
Analysis of the ROC curve for the identification of pulmonary fibrosis progression, stratified according to baseline infiltrated percentage.

Considering the 2022 ATS/ERS/JRS/ALAT guideline criteria, 26 (70.27%) out of 37 patients no longer met the definition for disease progression. We also performed a ROC analysis to evaluate the ability of the pre-treatment infiltrated volume (%) to predict the occurrence of progression criteria during nintedanib treatment. The area under the ROC curve (AUC) was 0.767, indicating moderate discriminative ability of the baseline infiltrated percentage in identifying individuals at risk of pulmonary fibrosis progression on treatment (Figure 3). The optimal threshold identified using Youden’s index was 21.90%, which provided a sensitivity of 91.70% and a specificity of 68.00%. These findings suggest that patients with an infiltration burden greater than 21.90% are more likely to experience a disease progression, highlighting the prognostic relevance of radiological involvement in predicting functional deterioration.

Adverse events aligned with the known safety profile of nintedanib. Gastrointestinal issues were most common—diarrhoea in 48.65% and nausea/vomiting in 40.54%—and generally ranged from mild to moderate, occurring early after treatment began. Most were managed conservatively with supportive care; when necessary, brief treatment pauses or dose reductions to 100 mg twice daily enabled continued therapy with re-escalation as tolerated. Permanent discontinuation occurred in 3 patients (7.5%). Overall, the tolerability profile in our group reflects previous trial and real-world data and supports the feasibility of maintaining long-term treatment in routine practice.

## 4. Discussion

This study leverages AI-powered QCT to offer a granular, objective assessment of nintedanib’s impact on the lung parenchyma in a real-world cohort of PPF. Our findings present a complex and nuanced picture of therapeutic response, highlighting both the promise of advanced imaging biomarkers and the intricate, multifaceted pathophysiology of the disease [24,25,26]. We documented a statistically significant reduction in AI-quantified infiltration in the right lung, alongside a non-significant trend toward reduction in total lung infiltration. This side-specific signal is radiologically plausible. The right lung is larger and trilobar, so small, diffuse improvements can aggregate into a measurable volumetric change. By contrast, the left lung is smaller and lies adjacent to the heart; left-sided measurements are more prone to cardiac motion, beam-hardening, and juxtacardiac partial-volume effects, particularly in the lingula and peri-hilar regions. These factors introduce greater segmentation uncertainty and measurement variability on the left, which can blur subtle treatment effects in quantitative CT (QCT) metrics despite identical biology. These findings should be interpreted cautiously and validated in larger cohorts.

The “infiltration” class in our AI-powered QCT analysis, defined by Hounsfield Units in the range of −750 to −400 HU, is believed to encompass a spectrum of parenchymal abnormalities, including ground-glass opacities and fine reticular patterns. These features often represent areas of active inflammation, cellular infiltration, and early, potentially modifiable fibrotic deposition. Nintedanib, a small molecule tyrosine kinase inhibitor, exerts its anti-fibrotic effect by targeting key signalling pathways involved in fibroblast proliferation, migration, differentiation, and extracellular matrix deposition, including those mediated by fibroblast growth factor receptors (FGFR), platelet-derived growth factor receptors (PDGFR), and vascular endothelial growth factor receptors (VEGFR) [27,28,29]. It is therefore biologically plausible that the observed reduction in the QCT-defined “infiltration” volume reflects the drug’s primary mechanism of action: the successful suppression of these active fibroproliferative processes.

Our results fundamentally challenge the sufficiency of FVC as a standalone endpoint for assessing therapeutic response in both clinical practice and research settings. In our cohort, the stability of FVC over one year, if viewed in isolation, could be interpreted as therapeutic success or disease stabilisation. This finding is of critical importance, as FVC remains the primary endpoint in most clinical trials for anti-fibrotic therapies and the principal metric for clinical decision-making. It underscores the urgent need for more sensitive, multi-faceted assessment tools that can provide a more holistic view of the disease state. This study reinforces the substantial value proposition of AI-powered QCT as a critical tool for the objective and sensitive monitoring of fibrosing lung disease, a concept strongly supported by a growing body of the literature advocating for the use of automated quantitative methods. The key advantages demonstrated in our analysis are many-fold. First, AI-powered QCT provides a level of objectivity and reproducibility that is unattainable with traditional, semi-quantitative visual scoring of HRCT scans, which is known to be subject to significant inter- and intra-observer variability. Second, the granularity afforded by AI-powered QCT is a considerable advance. The ability to segment the lung into distinct tissue classes based on density—such as infiltrated, collapsed, emphysematous, and usually inflated lung—allows for a differential analysis of the therapeutic effect on various pathological components. This is a far more sophisticated approach than relying on global physiological measures, such as FVC, which represent the integrated output of the entire respiratory system and can be influenced by numerous factors. Finally, the sensitivity of AI-powered QCT may allow for the detection of subtle parenchymal changes that precede a functionally significant decline, potentially establishing it as an earlier and more responsive biomarker of treatment efficacy or failure.

However, the interpretation of our AI-powered QCT findings requires scientific rigour and caution. While our study provides a detailed, mechanistic view of radiological change in a small, well-characterised cohort, its clinical relevance can only be fully understood when placed in the context of larger, real-world effectiveness studies. The recent Italian, observational, multicentre study by Mondoni et al. provides the ideal clinical framework for this interpretation [30]. Mondoni and colleagues investigated the effectiveness of nintedanib in a cohort of 172 patients with PPF, assessing outcomes based on the cessation of progression according to established clinical criteria. They demonstrated that nintedanib is clinically effective in a large, heterogeneous, real-world PPF population. They reported that after one year of treatment, a substantial majority of patients—64.4% according to INBUILD trial criteria and 79.3% according to the 2022 ATS/ERS/JRS/ALAT guideline criteria—no longer met the definition for disease progression. Similarly, we found that 70.27% of patients did not show progression of lung fibrosis. Moreover, Narváez et al. confirmed that antifibrotic initiation was associated with a modest improvement in the trajectory of FVC and stabilisation in DLCO [31]. Our results support previous evidence that automated QCT detects clinically significant disease signals beyond visual scoring. Jacob et al. showed that in IPF patients, CALIPER-derived measures had a stronger correlation than visual scores with pulmonary function and identified pulmonary vessel volume as a structure–function marker lacking a visual counterpart [32,33]. Overall, these cross-sectional data substantiate the biological validity of our QCT endpoints. In a large registry cohort of IPF, Humphries et al. showed that baseline CT fibrosis extent, measured by data-driven texture analysis, was linked to the subsequent rate of FVC and DLCO decline and also stratified transplant-free and progression-free survival, with prognostic value independent of pulmonary function [34].

Perhaps the most significant contribution of our study is its exploration of baseline parameters to predict individual patient trajectories, representing a crucial step toward a more personalised medicine approach for PPF, rather than assessing average treatment effects. Our ROC analysis showed that a high baseline burden of total infiltration—specifically, a value greater than 21.90%—was a moderate predictor of experiencing disease progression. Patients who start with a high disease burden are already far along the fibrotic continuum. Even if nintedanib effectively reduces the rate of new infiltration, their lungs are so structurally compromised that even a slight absolute decline in function is more likely to cross the relative threshold for clinical progression. Their overall prognosis remains poor despite a measurable drug effect on one component of their disease. This suggests the existence of a potential “therapeutic window”, where patients with an intermediate level of active disease may derive the most significant net clinical benefit. While preliminary and in need of extensive validation, this 21.90% AI-powered QCT threshold represents a tangible first step toward a quantitative, imaging-based risk stratification tool. In the future, clinicians could potentially use baseline AI-powered QCT analysis to identify patients at high risk for progression despite standard therapy. These individuals might then be targeted for more frequent monitoring, more aggressive management of comorbidities, or prioritisation for enrolment in clinical trials of novel combination therapies. Currently, AI-powered QCT should be regarded as a complement to respiratory functional testing. Decisions to continue, modify, or switch antifibrotic therapy should be based on clinical status, lung function trends, imaging biomarkers, oxygen needs, and history of exacerbations.

The primary limitation of our study is related to its retrospective design, which inherently carries a risk of selection and information bias, as well as its small, single-centre cohort of 37 patients. This limits statistical power and broadens confidence intervals, increasing the possibility of effect overestimation for individual QCT markers (e.g., infiltrated volume). Furthermore, his single-arm design mirrors real-world treatment pathways and aims to reduce interpatient heterogeneity by using each participant as their own control.

## 5. Conclusions

In conclusion, this study demonstrates that AI-powered QCT analysis is a powerful tool for dissecting the complex and heterogeneous effects of nintedanib on the lung parenchyma in patients with PPF. Most importantly, this work presents preliminary but compelling evidence that baseline quantitative imaging can serve as a predictive biomarker to stratify patients and anticipate their clinical trajectory. The identification of a prognostic AI-powered QCT threshold, though requiring validation, paves the way for a future of more personalised and precise management of this devastating disease. The translation of these promising findings into routine clinical practice is contingent upon rigorous validation in prospective, multicentre studies, which should be a high priority for the respiratory research community. Taken together, our results underscore the utility of AI-derived radiologic biomarkers in detecting early treatment responses and potentially anticipating functional decline. While changes in FVC and DLCO remain cornerstone endpoints, radiologic features—particularly infiltrated volume—may offer additional prognostic information and refine risk stratification in PPF. In fact, rather than challenging the adequacy of FVC as a primary endpoint, our results emphasise the complementary role of QCT as an addition to functional testing. Larger, prospective, case-control studies are needed to verify whether QCT can consistently detect treatment-related changes that happen before or alongside spirometric decline, providing valuable insights into long-term outcomes and modifications in follow-up HRCT.

## Figures and Tables

**Table 1:** Volumetric analysis parameters before and after 12 months of treatment with nintedanib.

Volumetric Analysis Parameter	Baseline	Follow-Up	*p*-Value
Infiltration right, mean value ± SD, cm^3^	5.56 ± 3.08	4.88 ± 2.77	0.041
Collapsed right, mean value ± SD, cm^3^	1.79 ± 1.24	1.77 ± 1.61	0.919
Vessels right, mean value ± SD, cm^3^	0.28 ± 0.27	0.33 ± 0.54	0.483
Infiltration left, mean value ± SD, cm^3^	4.73 ± 2.35	5.03 ± 3.09	0.555
Collapsed left, mean value ± SD, cm^3^	1.62 ± 1.50	1.77 ± 1.73	0.466
Vessels left, mean value ± SD, cm^3^	0.21 ± 0.27	0.36 ± 0.70	0.174

**Table 2:** Extended analysis parameters before and after 12 months of treatment with nintedanib.

Extended Analysis Parameter	Baseline	Follow-Up	*p*-Value
Total, mean value ± SD, mL	41.93 ± 1.21	41.85 ± 1.68	0.745
Functional, mean value ± SD, mL	22.62 ± 5.22	22.79 ± 5.87	0.800
Functional, mean value ± SD, %	53.90 ± 11.90	54.19 ± 13.30	0.855
Infiltrated, mean value ± SD, mL	10.30 ± 5.11	9.50 ± 4.73	0.199
Infiltrated, mean value ± SD, %	24.60 ± 12.26	22.81 ± 11.38	0.215
Affected, mean value ± SD, mL	13.60 ± 7.00	12.88 ± 6.80	0.400
Affected, mean value ± SD, %	32.51 ± 16.83	31.14 ± 17.03	0.510
Total right, mean value ± SD, mL	21.22 ± 0.69	21.01 ± 0.97	0.335
Functional right, mean value ± SD, mL	13.86 ± 4.14	5.98 ± 10.41	0.156
Functional right, mean value ± SD, %	65.49 ± 19.13	68.62 ± 18.41	0.128
Infiltrated right, mean value ± SD, mL	5.56 ± 3.08	4.88 ± 2.77	0.042
Infiltrated right, mean value ± SD, %	26.33 ± 14.55	23.31 ± 13.17	0.054
Affected right, mean value ± SD, mL	7.25 ± 4.04	6.53 ± 3.72	0.084
Affected right, mean value ± SD, %	34.51 ± 19.13	31.38 ± 18.41	0.128
Total left, mean value ± SD, mL	20.82 ± 0.99	20.84 ± 1.03	0.944
Functional left, mean value ± SD, mL	14.47 ± 3.78	14.48 ± 4.10	0.982
Functional left, mean value ± SD, %	69.35 ± 17.09	69.32 ± 18.57	0.100
Infiltrated left, mean value ± SD, mL	4.73 ± 2.35	4.63 ± 2.36	0.774
Infiltrated left, mean value ± SD, %	22.79 ± 11.40	22.30 ± 11.33	0.754
Affected left, mean value ± SD, mL	6.34 ± 3.51	6.35 ± 3.76	0.985
Affected left, mean value ± SD, %	30.63 ± 17.12	30.68 ± 18.60	0.128

**Table 3:** Lung functional parameters before and after 12 months of treatment with nintedanib.

Lung Functional Parameter	Baseline	Follow-Up	*p*-Value
RV, mean value ± SD, L	1.49 ± 0.56	1.29 ± 0.63	0.104
RV, mean value ± SD, %	60.96 ± 21.55	52.65 ± 24.61	0.093
FVC, mean value ± SD, L	2.58 ± 1.06	2.50 ± 0.82	0.469
FVC, mean value ± SD, %	72.68 ± 23.84	72.23 ± 16.53	0.885
FEV_1_, mean value ± SD, L	2.14 ± 0.83	2.06 ± 0.64	0.344
FEV_1_, mean value ± SD, %	78.82 ± 25.80	78.36 ± 18.66	0.894
PEF, mean value ± SD, L/s	7.35 ± 2.98	6.79 ± 1.82	0.300
PEF, mean value ± SD, %	101.80 ± 33.21	96.59 ± 20.92	0.447
FEF_25–75_, mean value ± SD, L/s	2.38 ± 1.18	2.37 ± 0.78	0.982
FEF_25–75_, mean value ± SD, %	115.00 ± 64.19	120.00 ± 45.09	0.563
DLCO, mean value ± SD, mmol/min/kPa	3.26 ± 1.21	2.76 ± 1.13	0.097
DLCO, mean value ± SD, %	43.06 ± 15.03	36.75 ± 12.68	0.096

## Data Availability

All data in support of the findings of this paper are available within the article.
